# Training in recovery, perfusion and packaging of organs for transplants: profile of professionals and analysis of post-course learning

**DOI:** 10.31744/einstein_journal/2019AO4445

**Published:** 2019-03-08

**Authors:** Juliana Guareschi dos Santos, Veronica Schonfeld Gomes Silva, Luciana Cintra, Cassiane Dezoti da Fonseca, Luciana Carvalho Moura Tralli

**Affiliations:** 1 Hospital Israelita Albert Einstein, São Paulo, SP, Brazil.; 2 Faculdade Israelita de Ciências da Saúde Albert Einstein, São Paulo, SP, Brazil.; 3 Escola Paulista de Enfermagem, Universidade Federal de São Paulo, São Paulo, SP, Brazil.

**Keywords:** Learning, Tissue and organ harvesting/education, Patient care team, Aprendizagem, Coleta de tecidos e órgãos/educação, Equipe de assistência ao paciente

## Abstract

**Objective:**

To understand the profile of professionals working in organ harvesting, and analyze the learning results of those trained before and after the course on recovery, perfusion and packaging of organs for transplants.

**Methods:**

A retroprospective, quantitative, analytical-descriptive study about the Course on Recovery, Perfusion and Packaging of Liver and Kidney, in the period from 2012 to 2014. Pre- and post-tests, with ten questions were used to assess knowledge about organ harvesting. The association of knowledge with applied content was verified by the McNemar test.

**Results:**

Of the total of 334 participants, 187 (56.0%) were physicians, 104 (31.1%) nurses, and 43 (12.9%) scrub nurses. The majority of participants was male (58.4%), mean age of 39.1 years, 50% had graduated 5 to 10 years before, and 50.4% had less than one-year experience in organ harvesting. In knowledge assessment, there was an increase in the weighted mean, from 6.1 in the pre-test to 7.9 in the post-test. A significant increase in learning was observed in the post-test in 50% of scrub nurses, 33.3% in nurses 20% in physicians.

**Conclusion:**

The professionals were starting work in organ harvesting, and most were from Southeastern, Northeastern and Northern regions. In terms of learning, the course contributed to enhancing knowledge of the multiprofessional health team, and represented better learning standard.

## RESUMO

**Objetivo:**

Conhecer o perfil dos profissionais que atuam em captação de órgãos e analisar o resultado da aprendizagem daqueles treinados antes e após o curso de extração, perfusão e acondicionamento de órgãos para transplantes.

**Métodos:**

Estudo retroprospectivo, quantitativo, analítico-descritivo do Curso de Extração, Perfusão e Acondicionamento de Fígado e Rim, no período de 2012 a 2014. Utilizaram-se o pré e o pós-teste estruturado em dez questões, que avaliaram o conhecimento sobre captação de órgãos. A associação do conhecimento com o conteúdo aplicado foi verificada pelo teste McNemar.

**Resultados:**

Do total de 334 participantes, 187 (56,0%) eram médicos, 104 (31,1%) enfermeiros e 43 (12,9%) instrumentadores. Houve predominância do sexo masculino (58,4%), com média de idade de 39,1 anos. Tinham entre 5 a 10 anos de formados 50% da amostra, e 50,4% tinham menos de 1 ano de experiência na área de captação de órgãos. Na avaliação do conhecimento, houve elevação na média geral ponderada de 6,1, no pré-teste, para 7,9, no pós-teste. Observou-se aumento significativo da aprendizagem no pós-teste em 50% nos instrumentadores, 33,3% nos enfermeiros e 20% nos médicos.

**Conclusão:**

Os profissionais eram iniciantes na área de captação de órgãos e, em sua maioria, oriundos das Regiões Sudeste, Nordeste e Norte. No quesito de aprendizagem, o curso contribuiu para o aumento do conhecimento da equipe multiprofissional em saúde, representando ganho no padrão de aprendizagem.

## INTRODUCTION

Liver and kidney diseases are chronic conditions that can progress to terminal stages, resulting in high mortality rates. Renal and hepatic replacement therapies, by means of organ transplants, offer increased survival, and which in most cases are the only therapeutical option.^(^ ^1^ ^)^

In 2017, more than 8 thousand solid organ transplants were performed, according to the *Registro Brasileiro de Transplante* [Brazilian Transplant Registry].^(^ ^2^ ^)^ Despite efforts of the *Associação Brasileira de Transplante de Organs* (ABTO) [Brazilian Association of Organ Transplants] to increase this figure with campaigns that raise awareness of the population as to the importance of organ donation, it still is insufficient to meet the demand of more than 23 thousand adult patients that make up the national solid organ transplant waiting list, which demonstrates the mismatch that exists between supply (inadequate number of organs), demand (high number of patients on the waiting list),^(^ ^3^ ^,^ ^4^ ^)^ and result (conversion rate of possible donors to potential donors).^(^ ^5^ ^,^ ^6^ ^)^

One of the rationales for this imbalance demonstrated by ABTO is the high rate of refusal (42%) in donating organs by family members.^(^ ^3^ ^)^

The primary factors that contribute towards the increased rates of family refusal for donation of organs are lack of knowledge about diagnosis of brain death (BD); lack of awareness of the deceased person’s wishes; inappropriate interview of Family members when requesting donation; problems with integrity or image of the body after removal of organs and tissues; religious issues, and refusal, in life, of the deceased. Moreover, other stressors, such as dissatisfaction with care received; receiving the news of brain death in an unsettled way, and delay in delivering the corpse.^(^ ^7^ ^,^ ^8^ ^)^

A study with 55 nurses and nurse technicians showed the difficulties in approaching the potential donor (PD) are associated with the lack of preparation of the nursing team (34.6%), followed by lack of materials (23.1%), inadequate structure (19.2%), delay in starting the protocol to confirm BD (11.6%), family refusal (7.7%), and insufficient team (3.8%).^(^ ^9^ ^)^

Within this context, the lack of experience of the multiprofessional team in harvesting and donating organs is the result of the generalist education in health-related undergraduate courses. Up to 92% of nursing and medical undergraduate students are unaware of the *Organização de Procura de* Órgãos *e Tecidos* , [Organ and Tissue Procurement Organization],^(^ ^9^ ^)^ and only 34% of medical undergraduate students in a rotation in intensive care (ICU) reported having assessed a patient with BD.^(^ ^10^ ^)^

Considering the need to perform studies that verify the performance of the multiprofessional team during the organ harvesting stages, it is crucial to assess knowledge of these professionals, by providing training at organizations and based on the pre- and post-tests.^(^ ^11^ ^)^

## OBJECTIVE

To understand the profile of professionals who working in organ harvesting, and analyze the result of trainee learning before and after a course on recovery, perfusion and packaging of organs for transplants.

## METHODS

This is a retrospective, quantitative, analytical and descriptive study, approved by the Research Ethics Committee of a philanthropic hospital, opinion no. 1.573.585, CAAE: 55480616.1.0000.0071. It analyzed the profile and pre- and post-test grades of the professionals participating in the Course on Recovery, Perfusing and Packaging of Liver and Kidney. A total of 17 courses were given, in that, 4 in 2012, 6 in 2013, and 7 in 2014. Each course had 21 vacancies, 12 for surgeons (hepatologists or urologists), 6 nurses, and 3 scrub nurses. The *Sistema Nacional de Transplantes* (SNT) [National Transplant System] directed the 357 vacancies toward all the State Centers for Reporting, Harvesting and Distribution of Organs and Tissues for Transplants, which indicated the candidates involved with harvesting, after selection by SNT in the Transplant Qualification Program. Furthermore, the system organized the logistics and methodology of the course via *Programa de Desenvolvimento Institucional do Sistema* Único *de Saúde* (PROADI-SUS) [Institutional Development Program of the Unified Health System]; and application was made online when the applicant answered the profile questionnaire that would be associated with the level of learning.

The total load was 16 hours, divided into 2 days. On the first day, the pre-test was applied, with ten multiple choice questions and four options of specific answers for each category, prepared by specialists in donations and transplants of the medical and nursing areas ( Appendix A ). Next, three lecture classes were given (surgical techniques of liver and kidney removal, and the role of the nurse as operating room coordinator). At the last class, the logistics of the harvesting process, printed material required by law, and aspects of organ packaging and transport were taught, based on the basic guidelines for harvest and removal of multiple organs and tissues,^(^ ^12^ ^)^ and on the *Resolution de Diretoria Colegiada* (RDC) [Collegiate Board Resolution] no. 66, of December 21, 2009.^(^ ^13^ ^)^

The practical class had a load of 13 hours. Nine female pigs were used, according to the norms and regulation of the Ethics Committee and the *Manual de Cuidados e Procedimentos com Animais de Laboratório* (CEUA: 2110_14) [Manual of Care and Procedures with Laboratory Animals]. On each operating table, the team simulated a surgery to recover liver and kidney. After the end of the practical phase, the students did the post-test, containing the same questions as the pre-test.

The Statistical Package for the Social Sciences (SPSS), version 17 (Chicago, Il, USA) was used. The scores were calculated by the total number of correct answers weighted by the number of valid questions answered by the professional, using the formula (number of correct questions)×10/number of valid questions.

Categorical variables were described by absolute frequencies and percentages, and the numerical variables, by means and standard deviations (SD) or medians. The general linear model was adjusted for the variable response, absolute difference between the pre- and post-test scores and the explanatory variable. The results of the models were presented by adjusted mean values and 95% confidence intervals, and the multiple comparisons were corrected by Bonferroni’s method. Association of knowledge of the professionals with the content applied was verified by the McNamara test.

## RESULTS

A total of 357 vacancies were provided, and 334 professionals attended the courses (23 were absent). The mean age was 39.1 years, and the standard deviation was 9.2 years. According to table 1 , 58.4% of professionals analyzed were males, 56% were physicians, 32% were from the Southeast Region, 74.5% had a specialization as additional training, and 98.8% had not attended courses geared toward the area of harvesting, recovery and packaging of organs within the previous 30 days.

**Table 1:** Sociodemographic characteristics of professionals

Variables	Total
	n (%)
Sex	
Female	139 (41.6)
Male	195 (58.4)
Professional category	
Nurse	104 (31.1)
Physician	187 (56)
Scrub nurse	43 (12.9)
Region	
North	58 (17.4)
Northeast	95 (28.4)
Central	40 (12.0)
Southeast	107 (32)
South	34 (10.2)
Further education	
None	56 (16.9)
Specialization	249 (74.5)
Master’s degree	20 (6.0)
PhD	8 (2.4)
Post-doctorate	1 (0.2)
In the last 30 days, have you attended any course related to donation-transplant process?	
Yes	4 (1.2)
No	330 (98.8)
Do you work directly in the organ donation process?	
Yes	135 (40.4)
No	199 (59.6)
Have you attended any multiple organ removal surgery?	
Yes	294 (88)
No	40 (12)
Do you take part in any organ harvesting team?	
Yes	123 (36.8)
No	211 (63.2)

The variable time since graduation showed the majority (50%; 167) had graduated between 5 and 10 years before, 29% (97) between 1 and 5 years before, 17.1% (27) more than 10 years, and 3.9% (13) less than 1 year.

As to practice in organ harvesting, 88% (294) of professionals had already watched organ removal surgeries, 40.4% (135) were working directly in the process of organ donation, and only 36.8% (123) were members of the organ harvest team.

Of the 135 professionals who were directly working in the donation process, 50.4% (68) had less than 1 year experience in this area, 35.6% (48) had between 1 and 5 years, 5.9% (8) between 5 and 10 years, and 8.1% (11) more than 10 years.

The profile variables (time since graduation, time in the area of donations, supplementary training, having watched an organ recovery surgery, and be a member of the harvesting team) did not show significant evidence.

Performance of students in the course was evaluated by the pre-test, with scores ranging from 1 to 10 (mean of 6.1 points; SD of 1.9) and by the post-test, with scores of 3 and 10 (mean of 7.9 pontos; SD of 1.4).

Physicians gained knowledge by 20%, the score rose from 6.6 to 8.3. Nurses had a 33.3% increase in score, from 5.5 going up to 7.4. The scrub nurses had their score of 4.9 enlarged to 7.4, inferring a 50% boost of knowledge after participation in the course.

The referred course favored a 31% increase in knowledge of the multiprofessional team as to organ harvesting.

Table 2 displays medical knowledge data regarding the aspects and attributions of surgeons and surgical techniques in the process of harvesting, removal and packaging of organs for transplants, per evaluated item in the pre- and post-tests. The items that showed significant correct answers were questions: 1, 3, 6, 7, 8, and 9. As to the post-course analysis, question 4 had the highest rate of errors, and all physicians answered question 5 correctly. There is no evidence of significant change in questions 2 and 10.

**Table 2:** Knowledge and changes in answers given by physicians for each question in the pre- and post-tests (n=187)

Topic of the question	Right answers		Wrong answers		Learning progression (%)	p value
	
	Pre-test	Post-test	Pre-test	Post-test		
Question 1: heparin dosage in organ recovery surgery	58	172	129	15	60.9	<0.001
Question 2: evaluation as to viability of the liver for transplant	130	137	57	50	3.7	0.066
Question 3: position of the cannula in perfusion relative to the renal arteries	152	181	35	6	15.5	<0.001
Question 4: previous actions of the harvest team for a safe organ recovery surgery	120	102	67	85	-9.6	0.007
Question 5: concept regarding ischemia time	165	187	22	0	11.7	
Question 6: aspects of exclusive renal removal	85	126	102	61	21.9	<0.001
Question 7: aspects related to kidney dissection	153	177	34	10	12.8	<0.001
Question 8: participation of the surgeon regarding inadequate perfusion	117	157	70	30	21.3	<0.001
Question 9: liver removal surgery and filling of the perfusing equipment	145	172	42	15	14.4	<0.001
Question 10: preservation solution used in the kidney machine*	11	13	24	22	1	0.234

* Blank items were not considered.

Table 3 shows how nurses were evaluated as to aspects of documentation, perfusion techniques, packaging, and transport of the removed organs, pre- and post-test, per item assessed. Question 7 was the only one that showed no evidence of significant change.

**Table 3:** Knowledge and changes in answers given by nurses for each question in the pre- and post-tests (n=104)

Topic of the question	Right answers		Wrong answers		Learning progression (%)	p value
	
	Pre-test	Post-test	Pre-test	Post-test		
Question 1: checking of documentation for safe organ removal surgery	77	90	27	14	12.5	0.005
Question 2: removal sequence of multiple organs	42	88	62	16	44.2	<0.001
Question 3: concepts about ischemia time	66	82	38	22	15.3	0.004
Question 4: organ identification label	52	73	52	31	20.1	0.001
Question 5: thermal coldbox-transport	73	86	31	18	12.5	0.011
Question 6: organ packaging	69	92	35	12	22.1	<0.001
Question 7: organ preservation solutions	35	43	69	61	7.6	0.054
Question 8: recovery and packaging of kidneys *en bloc* *	38	50	40	28	11.5	0.017
Question 9: participation of the perfusionist	82	94	22	10	11.5	0.003
Question 10: vessels that will be cannulated in organ perfusion	34	49	70	55	14.4	0.004

* Blank items were not considered.

Table 4 demonstrates the number of pre- and post-test correct answers, and the percentage of learning of each question for the scrub nurse as to the instruments used in removal surgery and cannulations, preservation solutions, and packaging. Significant correct answers were noted for questions 1, 2, 3, 4, 5, and 8. All scrub nurses answered question 9 correctly in the post-test. There was no evidence of significant change in questions 6, 7, and 10.

**Table 4:** Knowledge and changes in answers given by scrub nurses for each question in the pre- and post-tests (n=43)

Topic of the question	Right answers		Wrong answers		Learning progression (%)	p value
	
	Pre-test	Post-test	Pre-test	Post-test		
Question 1: multiple organ removal sequence	10	32	33	11	51.1	<0.001
Question 2: organ removal surgical times	12	19	31	24	16.2	0.047
Question 3: material used for cannulation of the arteries and veins in the removal surgery	14	38	29	5	55.8	<0.001
Question 4: pre-cannulation procedures	31	37	12	6	13.9	0.044
Question 5: solution used to cool the abdominal cavity at the time of removal	22	38	21	5	37.2	<0.001
Question 6: liver removal surgery and filling of perfusion equipment*	19	31	13	1	27.9	<0.001
Question 7: immediate cooling of the abdominal cavity	25	31	18	12	13.9	0.071
Question 8: organ packaging	16	24	27	19	18.6	0.033
Question 9: instruments used for removal surgery	40	43	3	0	6.9	
Question 10: participation of the scrub nurse at the time of donor exsanguination	17	19	26	24	4.6	0.175

* Blank items were not considered.

## DISCUSSION

National and international societies responsible for the organ donation-transplant process have established incentives, through public partnerships, with universities or charity hospitals that have projects to perform training and education development of healthcare teams regarding donation, harvest and transplants of organs. In this study, the partnership between the Ministry of Health and the Proadi-SUS^(^ ^14^ ^)^ reflected the significant presence of physicians, nurses, and scrub nurses who work directly or indirectly with organ donation or harvesting, in the Course for Recovery, Perfusion, and Packaging of Liver and Kidney. The reason is the public agencies that indicated these professionals understand that, by means of this qualification, it is possible to improve the rates of harvested and removed organs; and when they are available, mortality and morbidity rates of patients in the waiting list for transplants in their state or region will decrease. Additionally, it is possible to infer that the massive presence of these professionals in the course is due to the fact this topic is not addressed in undergraduate and graduate syllabuses of health-related courses in the country.^(^ ^13^ ^,^ ^15^ ^,^ ^16^ ^)^ Therefore, these professionals seek constant updating and training courses to enhance their knowledge, skills, attitude and current performance. In healthcare, knowledge and technology change at fast pace.^(^ ^17^ ^)^

Aiming at updates, this study addressed questions to meet the needs of the current scenario and of the participants. One example is discussing about systemic heparinization, which is widely used during donor’s hepatectomy. It is important to learn about this theme, due to the association between the heparin dose in the donor and the frequent occurrence of thrombosis in the vascular graft, which leads to future complications ine recipients.^(^ ^18^ ^-^ ^20^ ^)^

Among the topics covered for nurses and scrub nurses, the current norms and regulations, as per the items in RDC 66/2009 for optimization and legalization of the organ harvesting process^(^ ^21^ ^)^ in the perioperative period, showed an increase of learning rate. This result is similar to the findings of an investigation that concluded the factors interfering in the excellent quality of care delivered intraoperatively, in organ donation and harvest related-procedures, were level of knowledge and experience of nurses, minimizing adverse events regarding quality of organs and recovery of their recipients.^(^ ^15^ ^)^

The course also approached the use of active methodology and availability of vacancies nation-wide.

Simulation of organ recovery surgery in animals became an active strategy, and could be one of the contributing factors for increased learning performance for all categories after the course. According to a survey carried out with nurses and medical undergraduate students, the use of the simulation strategy made the participants acquire knowledge by 10% and 19%, respectively.^(^ ^22^ ^,^ ^23^ ^)^

Nevertheless, some contents applied had gaps in knowledge: for physicians, evaluation of viability of liver for transplantation, and preservation solution used in the kidney machine; for nurses, vessels that will be cannulated in perfusion of the organ; and for scrub nurses, filling of the perfusion lines, immediate cooling of abdominal cavity, and participation at the time to exsanguinate the donor. These were themes would improve the course, and must be reviewed and revalidated by specialists in the field, since they are fundamental for a safe process and quality of care delivered to the organ donor and recipient.

As to national distribution of vacancies, it was possible to decentralize knowledge and public investment. On the other hand, the course had a higher percentage of participants from the Southeast Region, and lower from the North Region, which could benefit if the selection criteria were based on State donor and transplant indicators.^(^ ^24^ ^)^ For example, the donor rate per million inhabitants (ppm) for the North Region was 3.9ppm, in 2017, and 17.9ppm for the Southeast Region. This 21% difference could be reduced with a greater distribution of education incentives for regions with lower rates, if the selection criteria for candidates for the next courses be changed.

The absence of a post-course support service to evaluate the impact of training in increased numbers of donors and organ harvesting in the country was a limiting fator, as well as the absence of more comparative studies regarding harvest, recovery, and packaging of organs for transplants. The review carried out between 1985 and 2013 showed that donation of organs had the highest percentage among the searched themes (86.2%), with 214 articles analyzed – in that, 73% about deceased donors, 15% living donors, and 10% donations in general. Nonetheless, the stage of organ harvesting was not specifically identified.^(^ ^10^ ^)^

The challenges to increase the quantity and improve the viability of organs harvested include maintaining campaigns with national scope, such as those launched by the Ministry of Health and by ABTO, which aim to raise awareness of the population about the altruism of donating organs, and its importance for the quality of life of recipients. Still, the use of technology for continued and tutored post-course qualification, such as telemedicine by means of video conferences, would be a useful instrument to address the factors contributing towards the challenges of the organ harvesting process. It can be employed both in distance education, for updating professionals at a lower cost, as well in administration and management of problems, by means of real-time communication through scientific and interdisciplinary sessions, and by prompt service in cases of healthcare process requirements.^(^ ^25^ ^,^ ^26^ ^)^

## CONCLUSION

Knowledge applied in the course showed increased learning for all categories, and it was more significant for scrub nurses and nurses. For physicians, a high level of previous knowledge was observed, considering the pre-test score higher than the total score of the course. A few knowledge gaps were observed for all categories. No significant evidence was found in the association between the variables of the sample profile and learning.

## Appendix A

**Table:** Questions and answer alternatives related to the pre- and post-test applied per professional category

Physician	Nurse	Scrub nurse
1. In the organ recovery surgery, what is the heparin dose? a) 100IU/kg. b) 200IU/kg. c) 300IU/kg. d) 400IU/kg.	1. Tick which documents should be pre-checked for organ recovery surgery: a) Declaration of brain death, report of supplementary exam/test for diagnosis of brain death, consent form for donation of multiple organs, information file of the donor of multiple organs, and serology and blood typing results. b) Report of supplementary exam/test for diagnosis of brain death. c) Only registration form at the organziation. d) Nothing.	1. What is the sequence for removing multiple organs? a) Lungs, heart, pancreas, liver, intestines, and kidneys. b) Heart, lungs, liver, kidneys, pancreas, and intestines. c) Heart, lungs, liver, pancreas, intestines, and kidneys. d) Heart, lungs, liver, pancreas, kidneys, and intestines.
2. Based on the characteristics below, which is not considered decisive for the viability of a liver transplant? a) Color. b) Consistency. c) Surface aspect. d) Anatomy of the hepatic artery.	2. What is the sequence for removing multiples organs? a) Lungs, heart, pancreas, liver, intestines, and kidneys. b) Heart, lungs, liver, kidneys, pancreas, and intestines. c) Heart, lungs, liver, pancreas, intestines, and kidneys. d) Heart, lungs, liver, pancreas, kidneys, and intestines.	2. What are the surgical times that make up the organ recovery surgery? a) Incision, inspection of cavity, warm dissection, cannulation, *in situ* perfusion, cold dissection, hepatectomy, table perfusing, removal of vascular and arterial grafts, and packaging of the organ. b) Incision only. c) Incision, removal of vascular and arterial grafts, and packaging of the organ. d) Exclusive packaging of the organ.
3. What is the position of the cannula in perfusion relative to the kidney arteries? a) Higher. b) Same height. c) Lower. d) Lateral.	3. How is warm ischemia time defined? a) From the time of clamping to the reperfusion of the organ in the recipient. b) From the time of clamping to the removing the graft from ice (in the operating room of the transplanting hospital). c) From the removal of the donor to ice. d) Nothing.	3. Which material can be utilized for the cannulation of these arteries and veins? a) Foley tube. b) Nasogastric tube. c) Orotracheal tube. d) Nelaton tube. e) Nothing.
4. Before initiating the organ recovery surgery, the harvest team should not: a) Check the laboratory tests, ABO typing, and serology of the donor. b) Check the conformity with the brain death protocol and the terms of donation. c) Collect blood sample to perform new laboratory tests. d) Evaluate the hemodynamic stability of the donor (number and doses of vasoactive drugs).	4. According to Anvisa Resolution RDC 66/09, which items should be on the organ identification label that will be attached to the organ’s packaging? a) RGCT of the donor, type of organ, and initials of the donor. b) RGCT of the donor, type of organ, and name of the donor. c) RGCT of the donor, type of organ, and laterality. d) RGCT of the donor, type of organ, and destination.	4. Which procedures come before cannulation? a) The choice of cannula number. b) Cannula connection on the perfusion lines. c) Filling of the perfusion lines. d) All of the alternatives (a, b, and c).
5. When does the ischemia time of an organ for transplant start? a) When the perfusion of the organ is concluded. b) When the organ is removed from the donor. c) When the aorta is clamped. d) When the organ is removed from ice.	5. According to Anvisa RDC 66/09, among the items that should appear on the identification label that will be attached to the coldbox are: a) RGCT and the donor’s hospital registry. b) Name of the origin depatment and of the sender. c) Name of the destination depatment and of the addressee. d) All of the above.	5. What is the solution used to cool the abdominal cavity when removing multiple organs for transplant? a) Frozen 0.9% saline solution. b) Frozen 5% glucose solution. c) Cold 0.9% saline solution (2 to 8ºC). d) Cold 5% glucose solution (2 to 8ºC).
6. As to exclusive kidney removal, tick the correct alternative: a) The cannula is inserted in the aorta, above the celiac trunk. b) One cannula is positioned in the aorta and another in the portal vein. c) Perfusion is carried out directly in the bilateral renal veins and arteries. d) Nothing.	6. Considering the packaging of organs for transplants, according to the Anvisa RDC 66/09, the first package contains: a) Sterile solution in a volume sufficient to protect the organ from external shocks. b) The organ and the preservation solution, and capacity proportional to the volume of the organ to be packaged. c) Ice (melting point at 0°C). d) Nothing.	6. What should the scrub nurse assure during the liver removal surgery? a) The presence of air in the perfusion lines. b) Absence of air in the perfusion lines. c) The presence of glucose solution in the perfusion lines. d) The presence of Ringer lactate solution in the perfusion lines.
7. Among the alternatives during dissection of the kidneys, it is importante to: a) Maintain them and send them *en bloc* to the transplant team that will separate them. b) Maintain the perfusion tube in the infrarenal vena cava. c) Maintain the periureteral fat so as not to compromise the irrigation of the ureter. d) Remove the fat to visualize the aspect of the organ.	7. Which organ preservation solutions can be used in the *in situ* perfusion of abdominal grafts? a) Only Wisconsin (Belzer^®^) solution. b) Euro-Collins. c) IGL 1 and Soltran. d) Saint Thomas.	7. What step should be taken to guarantee immediate cooling of the cavity? a) Approximation of the basin containing cold 0.9% saline solution, to the operative field. b) Approximation of the basin containing frozen 0.5% glucose solution, to the operative field. c) Approximation of the basin containing frozen 0.9% saline solution, to the operative field. d) Approximation of the basin containing cold 0.5% glucose solution to the operative field.
8. How should the surgeon proceed if the kidneys are poorly perfused? a) Perform reperfusion with cold 0.9% saline solution. b) Perform reperfusion with preservative solution. c) Package and document it on the operative description. d) Invalidate the organ for transplantation.	8. The recovery and delivery of kidneys *en bloc* should be done mandatorily in which situation below (ordinance no. 2.600/2009)? a) According to the team’s decision. b) In all donors. c) Pediatric deceased donor with weight ≤15kg or age ≤3 years. d) Pediatric deceased donor with weight ≤15kg or age ≤10 years.	8. Considering the packaging of organs for transplants, according to the Anvisa RDC 66/09, the first package contains: a) Sterile solution in a volume sufficient to protect the organ from external shocks. b) Organ and preservation solution, besides having the capacity proportional to the volume of the organ to be packaged. c) Ice (melting point 0°C). d) Nothing.
9. Considering packaging of organs for transplants, and according to the Anvisa RDC 66/09, the first package contains: a) Sterile solution in a volume sufficient to protect the organ from external shocks. b) Organ and the preservation solution, and have a capacity proportional to the volume of the organ to be packaged. c) Ice (melting point 0°C). d) Nothing.	9. During the perfusion, which items should the perfusionist observe and report to the surgical team: a) Infusion time and volume infused, in addition to possible complications. b) Infusion time, infusion rate, volume infused, and possible complications. c) Nothing should be observed. d) Report only the complications.	9. Which set of instruments is used for multiple organ surgery? a) Abdominal surgery box + vascular box + sternal saw + abdominal retractor + Finochietto retractor. b) Vascular box + small surgery box. c) Urology box + small surgery box + sternal saw + Doyen retractor valve. d) Abdominal surgical box + sternal saw + abdominal retractor + Finochietto retractor.
10. What is the preservation solution used in the kidney machine? a) Eurocollins + cold saline + frozen saline. b) SPS1 + saline at room temperature + cold saline. c) Kps + crushed ice + 1L of water. d) Custodiol + frozen and crushed saline + 1L of water.	10. In liver recovery surgery, which are the vessels to be cannulated for the perfusion of the organ? a) Aorta and vena cava. b) Aorta and gastroduodenal artery. c) Splenic artery, left gastric artery and portal vein. d) Aorta and superior or inferior mesenteric vein.	10. What should be done at the time of donor exsanguination? a) Request several packages of compresses. b) Request two suction devices that function well. c) Request two suction devices, but only one of them is working. d) Request two potent aspirators and reserve suction flasks in the room.
